# P-848. Proactive Pharmacy-led Transition of Care Protocol to Increase Appropriate Oral Antibiotic Use in Uncomplicated Gram-Negative Bacteremia

**DOI:** 10.1093/ofid/ofae631.1040

**Published:** 2025-01-29

**Authors:** Julia Kufel, Cadhan McFadden, Lisa Avery

**Affiliations:** St. Joseph's Health, Syracuse, New York; St. Joseph's Hospital, Syracuse, New York; St. John Fisher College Wegmans School of Pharmacy, Rochester, New York

## Abstract

**Background:**

Limited data are available to describe a pharmacist-driven Gram-negative bacteremia (GNB) protocol on treatment optimization and outcomes. The primary outcome was a composite of the percentage of patients on a pharmacist-driven antibiotic protocol. Secondary outcomes evaluated 30-day all-cause mortality and infection-related readmission.

**Methods:**

This was a single-center, quasi-experimental study conducted in a 451-bed community teaching hospital. The pre- and post-intervention cohorts included patients from 8/1/22-9/30/23 and 10/1/23-4/31/24, respectively. Patients aged >18 years with GNB were included. Patients who are immunocompromised, had a non-functional gastrointestinal tract, were enrolled in hospice, expired during hospitalization, pregnant, left against medical advice, had a polymicrobial infection, bacteria resistant to all PO agents, inadequate source control, or those with infectious disease consultations were excluded. For the intervention, a pharmacist-driven GNB protocol was developed to include the definition of uncomplicated GNB, clinical improvement criteria, ranked order of preferred PO antibiotics with appropriate dosing and duration of therapy. These recommendations were communicated to providers by pharmacists. The Chi-square test and student t-test were used for nominal and continuous data, respectively. A p-value of < 0.05 was considered statistically significant.

**Results:**

There were 61 and 41 patients in the pre- and post-intervention cohorts, respectively. Demographics were similar. The primary outcome was achieved in 59% and 81% of patients, respectively (p=0.02). Urinary source was most common, including 75% in the pre and 85% in the post-intervention group (p=0.22). Trimethoprim-sulfamethoxazole use increased from 6% to 29% (p=0.002). No difference in 30-day all-cause (p=0.51) and infection related readmission (p=0.82) was identified.

**Conclusion:**

Implementation of a proactive pharmacy-led GNB protocol increased the use of appropriate PO transition regimens in uncomplicated GNB.

**Disclosures:**

**All Authors**: No reported disclosures

